# Late Onset Combined Immunodeficiency Presenting with Recurrent *Pneumocystis jiroveci* Pneumonia

**DOI:** 10.1155/2014/801805

**Published:** 2014-03-31

**Authors:** Ilias Papakonstantinou, Ioannis G. Baraboutis, Lazaros Karnesis

**Affiliations:** ^1^1st Department of Internal Medicine, 401 General Military Hospital of Athens, Mesogeion Avenue 138, 11525 Athens, Greece; ^2^Infectious Diseases & HIV Division of the 5th Department of Internal Medicine, “Evangelismos” General Hospital, 45-47 Ipsilantou Street, 10676 Athens, Greece

## Abstract

Late onset combined immunodeficiency (LOCID) is a recently described variant of common variable immunodeficiency (CVID), involving adult patients presenting with opportunistic infections and/or low CD4+ lymphocyte counts. A 36-year-old male with unremarkable past medical history presented with fever, respiratory failure, and lymphocytopenia. He was found to have *Pneumocystis jiroveci* pneumonia (PJP), subsequently complicated by recurrent hospital-acquired *Pseudomonas aeruginosa* pneumonia and immune reconstitution phenomena, attributed to restoration of immunoglobulin levels. Clinicians should be aware of LOCID, which could be confused with HIV infection/AIDS or idiopathic CD4 lymphocytopenia. In the English bibliography there is only one case report, where PJP was the initial presentation of CVID (that case would probably be classified as LOCID). Phenomena of immune reconstitution are described in various settings, including primary immunodeficiency, manifesting as temporary clinical and radiologic deterioration and leading to misperceptions of therapeutic failure and/or presence of alternative/additional diagnoses.

## 1. Introduction

Common variable immunodeficiency (CVID), despite being a relatively rare condition, represents the most commonly encountered primary immunodeficiency in clinical practice associated with clinically significant antibody failure [1]. This intrinsic antibody deficiency may appear at any age and presents enormous heterogeneity [1, 2]. Malphettes et al. identified a subgroup of CVID patients enrolled in the DEFI cohort study, characterized by the occurrence of opportunistic infections (OIs) and/or a CD4+ T cell count of <200 cells/*μ*L, and showed significant differences from classic CVID patients in terms of clinical and immunologic characteristics. The authors introduced the term “late onset combined immunodeficiency” (LOCID) to describe this distinct CVID patient subgroup [3]. Similar observations have also been made by others [4]. We report the case of an adult male patient, who presented with* Pneumocystis jiroveci* (PJP) pneumonia and from further evaluation he eventually was found to fulfill LOCID criteria. His hospital course was complicated by recurrent* Pseudomonas aeruginosa* pneumonia and possible immune reconstitution phenomena.

## 2. Case Presentation

A 36-year-old Caucasian male was admitted to the hospital on August 2006 with a history of a 15-day low grade fever, weight loss, nonproductive cough, and progressive dyspnea. His previous personal and family history were unremarkable. He was not smoking and was not receiving any medications on a regular basis. Review of systems revealed frequent episodes of respiratory infections over the past 5 years without the need for hospitalization. Physical examination showed bilateral crackles and rales involving all lung fields bilaterally and no significant lymphadenopathy, hepatosplenomegaly, or any abnormalities from the skin and cardiovascular system.

**Figure 1:**
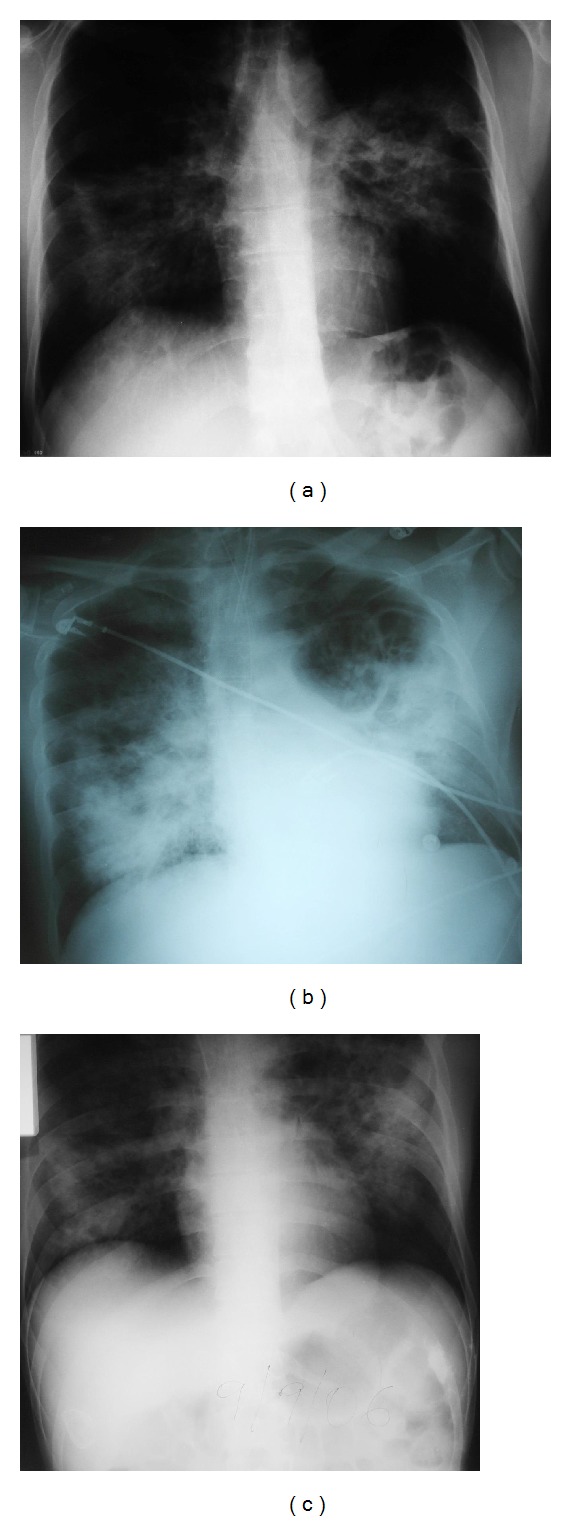
(a) Chest X-ray on admission with diffuse bilateral infiltrates. (b) 2 weeks after admission, intubated, receiving TMP/SMZ plus broad-spectrum antibiotics, after a 5-day immunoglobulin infusion course. First appearance of ground glass opacification and cystic/bullous lesions.* Pseudomonas aeruginosa* isolated from blood cultures. (c) 3.5 weeks after admission, short term clinical improvement.

The initial white blood cell (WBC) count was normal but severe lymphocytopenia (<200 cells/*μ*L) was noted. The patient deteriorated clinically and a decline of WBCs, platelet count, and hematocrit was noted while total lymphocyte count decreased to <60 cells/*μ*L. On presentation, the erythrocyte sedimentation rate and C-reactive protein were elevated and lactic dehydrogenase levels were 1485 U/L. Among other markers of inflammation, ferritin levels were within normal limits and the levels of fibrinogen were increased (687 mg/dL). Due to depression of all cell lines in the periphery, bone marrow biopsy was undertaken. Bone marrow was hypercellular with mild dysplastic changes, and it contained macrophages with increased phagocytic activity, revealing hemophagocytic syndrome. The initial chest X-ray (CXR) showed bilateral diffuse infiltrates arising from the perihilar regions (Figure 1(a)). Initial Gram stains and cultures from sputum were negative. At that point, antibiotics against pathogens of community-acquired pneumonia were administered and the patient underwent bronchoscopy.

While waiting for bronchoscopy results, he developed worsening hypoxemia and respiratory failure that required intubation and transfer to the hospital's intensive care unit (ICU). Bronchoalveolar lavage (BAL) analysis revealed* Pneumocystis jiroveci* (PJP) and high-dose trimethoprim-sulfamethoxazole (TMP-SMX) along with corticosteroids was added to broad-spectrum antibiotics. Examination for other pathogens, including* Mycobacterium tuberculosis*, atypical mycobacteria, cytomegalovirus (CMV),* Cryptococcus, *and* Aspergillus* species, was unrevealing.

The patient showed no significant clinical improvement. New blood and sputum cultures sent 5 days after his ICU admission revealed multiresistant* Pseudomonas aeruginosa* (*P. aeruginosa*), sensitive only to amikacin and colistin and intermediately sensitive to piperacillin-tazobactam. Colistin and amikacin were added to meropenem, intravenous pentamidine was added to TMP-SMX, and granulocyte colony stimulating factor was administered.

Laboratory evaluation for immunodeficiency had already begun. Serum antibody assays for HIV-1 and -2 were negative. Serum immunoglobulin levels were markedly reduced: IgM 0.259 g/L (normal range: 0.4–2.8 g/L), IgG 3.9 g/L (normal range: 7–17 g/L), IgG1 2.81 g/L, IgG2 0.426 g/L, IgG3 0.331 g/L, and IgG4 0.0198 g/L. Immunofixation that performed as a standard laboratory procedure, excluded a subsequent overproduction of an IgA monoclonal paraprotein. When those results became available, intravenous immunoglobulin infusions (IVIG) were administered, with the regimen Pentaglobin at a dose of 400 mg/kg/day for 5 days.

While the patient was receiving the above-described therapy and was showing minimal improvement, his CXR was evolving and showed ground-glass opacification and bullous-type lesions at the upper lung fields bilaterally (Figure 1(b)). The patient's clinical condition improved significantly almost 2 weeks after appropriate antimicrobial and adjunctive therapy (Figure 1(c)). At that time (approximately 4 weeks after the onset of respiratory failure), PJP full therapy was substituted by secondary prophylaxis and broad-spectrum antibiotics were discontinued.

**Figure 2:**
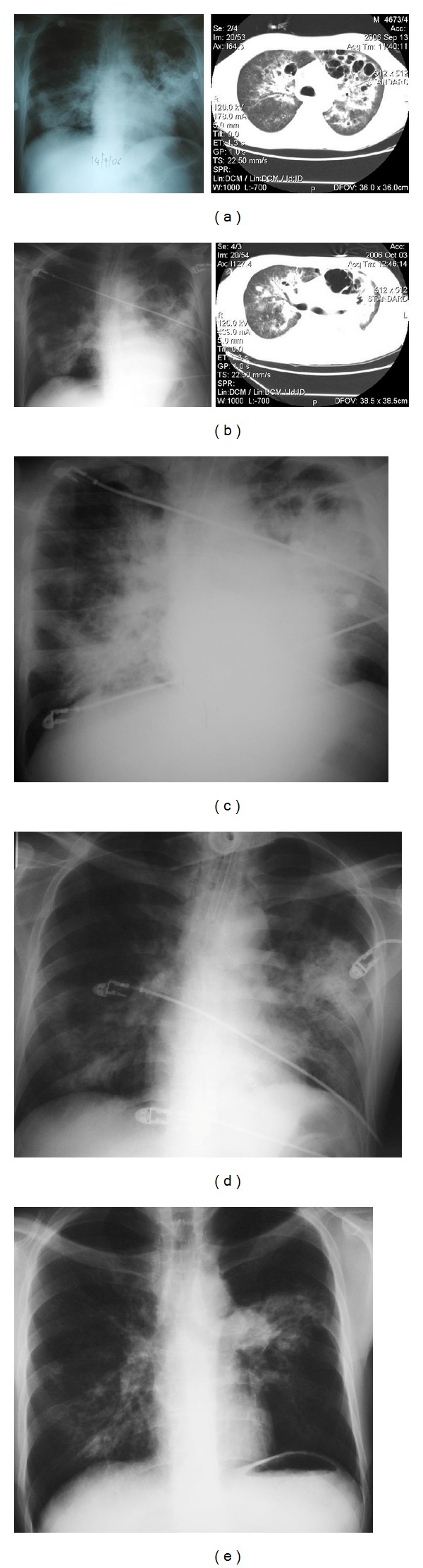
(a) 5 weeks after admission, new respiratory failure, bullous lesions, and dense infiltrates. (b) 7 weeks after admission, receiving again TMP-SMZ, now with clindamycin, and antipseudomonas antibiotics (colistin, ciprofloxacin, and imipenem), after the 2nd immunoglobulin infusion course. (c) 9 weeks after admission, radiologic deterioration of dense infiltrates.* Pseudomonas aeruginosa* still isolated from bronchial samples, no other pathogen identified. (d) 10 weeks after admission, significant clinical improvement. (e) 11 weeks after admission, before hospital discharge.

Three to four days later, the patient's respiratory status deteriorated again. New CXR and computed tomography (CT) scans revealed worsened dense infiltrates and bullous lesions (Figure 2(a)). Broad-spectrum antibiotics were reinstituted plus empiric antifungal and antituberculosis therapy. Repeated testing for* Mycobacterium tuberculosis*,* Mycobacterium avium* complex, and fungi via stains, cultures, and molecular detection by polymerase chain reaction (PCR) was negative. New bronchial cultures and BAL studies revealed the same pathogens—*Pneumocystis jirovecii* and* P. aeruginosa.* Anti-PJP therapy was reinstituted with TMP-SMX plus clindamycin at this stage, while empiric antifungal and antituberculous therapies were discontinued. Immunoglobulin infusions for 5 more days at the same regimen and dosage as previously were administered. Patient's CXR continued to evolve during therapy and* P. aeruginosa* was repeatedly isolated from bronchial secretions (Figures 2(b) and 2(c)). The patient responded clinically to his new regimen after 2-3 weeks and radiologically after almost 4-5 weeks (Figures 2(d) and 2(e)). Secondary PJP prophylaxis was again initiated.

Further investigations: due to history of recurrent respiratory tract infections and the newly discovered hypogammaglobulinemia, further analysis for suspected immunodeficiency was undertaken.

1. Immunoglobulin level serial measurements during hospitalization and follow-up are depicted on Table 1.
2. Post test immunization (*Haemophilus influenzae* type b, polysaccharide antigen of* Streptococcus pneumoniae*, and tetanus toxin) specific antigenic responses were absent, indicative of defective antibody production.
3. Adenosine deaminase (ADA) enzymatic activity in red blood cells was normal.
4. Tumour necrosis factor receptor superfamily member 13B (TNFRSF13B, also known as TACI) expression on *Β* lymphocytes, assessed by flow cytometry, was within normal range (8.7%) and no defect on TNFRSF13B gene encoding TACI protein was recognized by gene sequencing.
5. Lymphocyte proliferation in response to antigens (tetanus toxoid and* Candida*) was not impaired (impaired responses in 20–40% of CVID patients).
6. HLA-DR expression on CD3+ T lymphocytes (suggesting activation of T cells) was 56% soon after hospital discharge and 15.5% a few months later (measurements performed in different laboratories). Cytokines IL-2, IL-4, IL-5, IL-10, IL-12, and INF-*γ* levels were measured and were found to be within normal range.
7. Initial immunophenotyping revealed marked lymphopenia (total lymphocytes: 320 cells/*μ*L) and marked decreases of peripheral CD4+ T (16.4%), CD8+ T (8%), and CD19+ B lymphocytes (1.5%), while NK cell percentage was found elevated (60.3%). During follow-up, CD4+ T cells were persistently low, while CD8+ T cells and B cells gradually increased to normal levels, and NK cell percentage decreased to near-normal range. The percentage of naïve CD4+ T cells (CD4+ CD45RA+) remained persistently low (Table 2).
8. Repeated serum antibody assays for HIV-1 and -2 and HTLV I and II as well as quantitative HIV-1 plasma RNA measurement were negative.

Based on the diagnostic criteria proposed by the European Society for Immunodeficiencies and Pan-American Group for Immunodeficiency (age older than 4 years, serum IgG level below the lower reference range, decreased serum IgM or IgA, and exclusion of an underlying cause) [5], the patient's immune deficiency was labelled as CVID, while, according to the criteria proposed by Malphettes et al. [3], he was placed at the LOCID category/variant.

After hospital discharge, the patient had been regularly monitored and did not develop any other OIs. An additional bone marrow examination, performed almost one year after initial presentation, was within normal limits with slightly reduced bone marrow cells. He received secondary prophylaxis against PJP due to persistently low CD4 count and immunoglobulin substitution therapy periodically with subcutaneous injections. He developed a chronic diffuse eczema-type rash attributed to autoimmunity. He had a few bouts of diarrhea without documented infectious etiology that progressed over time and were attributed to small bowel enteropathy similar to celiac disease. Unfortunately the patient died, five years after the initial presentation to us due to unremitted intestinal disease that progressed to ileus, bowel perforation, and septic shock.

## 3. Discussion

According to the report by Malphettes et al. [3], LOCID patients may differ in several features from the rest of CVID patients. They may be characterized by a higher prevalence of splenomegaly, granuloma, gastrointestinal disease, and lymphoma and, even on immunoglobulin substitution, they may require more frequent antibiotics administration and hospitalization. The lymphocyte counts are characteristically lower, with a marked decrease in CD4+ T cell counts and a severe defect in naive CD45RA+ CCR7+ CD4+ T cell counts. The CD19+ B cell compartment may also be significantly decreased [3]. Our patient had a documentation of a persistently low CD4+ cell count, both during PJP and later, and had a very low naive CD45RA+ CCR7+ CD4+ T cell count and also initially depressed CD8+ T and CD19+ B cell counts. He did not have splenomegaly by clinical exam and he did develop gastrointestinal manifestations. Our patient at a later stage died by prominent complications of gastrointestinal disease. The natural course was in accordance with the observations by Malphettes et al. who noted that the subgroup of LOCID patients are more likely to have a severe clinical phenotype [3]. Among the gastrointestinal manifestations that frequently accompany CVID/LOCID patients, a large bowel enteropathy resembling inflammatory bowel disease and small bowel enteropathy that resembles celiac disease can develop [6].

Our patient presented with PJP as the initial manifestation of his immunodeficiency, even though, on further questioning, he did have frequent (more than 3/year), not clinically significant, respiratory tract infections, during the previous 4-5 years. In the first national immunodeficiency disease survey conducted in the United States, the most common diagnoses before the diagnosis of CVID were as follows (in order of frequency): sinusitis (67%), bronchitis (55%), pneumonia and ear infections (51% for each), diarrhea (30%), malabsorption (9%), sepsis (5%), meningitis (4%), hepatitis (3%), and cancer (2%) [7]. In a Brazilian cohort of 71 patients followed between 1980 and 2003, OIs appeared in 18% of patients, among which one case of probable (not definite) PJP was reported [8]. Analyzing 313 patients with CVID from the French DEFI study, Malphettes et al. found 17 patients (5.4%) who experienced opportunistic infections. There were 2 PJP cases that both occurred several years after CVID diagnosis (reported CD4 counts of 150 and 322 cells/*μ*L, resp.). In the first of them, the patient was receiving corticosteroids for lymphoma [3]. In a large US survey 13 of 248 patients with CVID (5.2%) experienced OIs during 25 years of observation [9]. Among them there were 7 cases of PJP (2.8%); the authors in this study did not clarify if PJP was the initial presentation of CVID [9]. We performed an additional English literature search to identify any reports of PJP as the initial presentation of CVID. One case report (published in 1994) described a patient with immunologic characteristics compatible with LOCID (low CD4 and CD8 count). In this case PJP was the initial clinical presentation, mimicking HIV infection [10].

Another clinical syndrome to be investigated at presentation, due to evidence of hemophagocytosis in the bone marrow, was Hemophagocytic lymphohistiocytosis (HLH), a syndrome of massive inflammatory response and elevated circulating cytokines [11, 12]. HLH onset in adults is uncommon [13] and also PJP can be rarely observed at the onset of HLH [14]. The diagnostic guidelines of HLH include a molecular diagnosis consistent with mutations in the perforin gene (PRF1), fever, splenomegaly, cytopenia, hypertriglyceridemia and/or hypofibrinogenemia (fibrinogen ≤150 mg/dL), hemophagocytosis in BM, spleen, or lymph nodes, low or absent NK-cell activity, ferritin ≥500 *μ*g/L, and elevated serum soluble CD25 that is soluble interleukin 2 (sIL2r) receptor [11, 13]. Although the molecular tests were not available to us during our patient's investigation, we advocate that the HLH syndrome was not applicable to our case because of ferritin's normal levels, increased levels of fibrinogen, absence of splenomegaly, and serum cytokines normal levels. The importance of HLH syndrome is mentioned, because of its ability to mimic many other common diseases. This should be taken into account, especially by the intensive care unit physicians, for patients who present unremitting severe sepsis and/or septic shock with cytopenia, elevated serum cytokines and ferritin levels, and histiocytic hemophagocytosis on bone marrow aspiration [12].

Regarding the principal differential diagnosis in our case, significant causes of adult onset immune dysfunction that had to be excluded were idiopathic CD4 lymphocytopenia (ICL) and adenosine deaminase (ADA) deficiency. Secondary etiologies associated with hypogammaglobulinemia, such as lymphoma, leukemia, thymoma, sarcoidosis, nephrotic syndrome, protein-losing enteropathy, and intestinal lymphangiectasia, were excluded during investigation [3, 6].

Idiopathic CD4+ lymphocytopenia (ICL) is a rare syndrome of severe CD4 T cells decrease in the absence of HIV infection or any defined immunodeficiency. Patients with ICL are reported to have reversal in CD4 : CD8 ratio, accompanied by reductions in the levels of several other lymphocyte subgroups and high or slightly low immunoglobulin levels [15].

Adenosine deaminase deficiency, a form of severe combined immunodeficiency (SCID), known as the T(−)B(−)NK(−) form of SCID, is a disorder of the purine salvage pathway that causes profound lymphopenia and molecular defects that lead to severe compromise in the number and function of T cells, of B cells, and occasionally of natural killer (NK) cells [16–18].

Combined immunodeficiency with impaired numbers and function of T and B cells can be attributed to defects in the recombinase activating genes (RAG) [19]. Hypomorphic variants of RAG genes with null mutations cause severe combined immunodeficiency (SCID) as was found in an increasing number of patients with combined immunodeficiency [20]. A case of adult onset idiopathic T cell lymphopenia due to recombinase activating gene 1 (RAG1) deficiency was also reported adding further questions to the impact of these hypomorphic mutations on immune function [21]. It is quite possible that patients classified as LOCID may indeed have mutations in RAG genes or other genes associated with combined immunodeficiency and in the future may be reclassified after genetic analysis.

Our patient's complicated clinical course, characterized by clinical deterioration and improvement, would not be uncommon or unexpected in an immunodeficient patient with severe structural lung damage, even despite appropriate treatment. Another possible hypothesis would be the attribution of concomitant immune reconstitution syndrome (IRIS). IRIS has been mostly described in advanced HIV infection after initiation of antiretroviral therapy [22] and also in other settings, such as BK virus infection in kidney transplantation [23], hepatitis B infection, and immunosuppression [24] after high-dose chemotherapy for leukemia [25], after mismatched hematopoietic stem cell transplantation [26], and so forth. Immune reconstitution phenomena have also been described with viral infections in severe combined immunodeficiency after bone marrow transplantation [24]. Such a syndrome could be defined as an acute symptomatic or paradoxical deterioration of a (presumably) preexisting infection that is temporally related to the immune system's recovery and could be misinterpreted as with therapy failure and/or presence of alternative/additional diagnoses [22, 24]. We could not find any reports in the English bibliography of IRIS on common variable immunodeficiency. At least part of patient's clinical and radiologic worsening during bouts of PJP and* P. aeruginosa* pneumonia could possibly be attributed to immune reconstitution, after immunoglobulin substitution, since the isolated* P. aeruginosa* strain maintained the same susceptibility pattern throughout. Additionally, with the exception of a few days after transient improvement, the patient was receiving appropriate antibiotic treatment and no other pathogens were subsequently isolated. Besides the expected beneficial effect of boosting immunoglobulin levels when facing a severe bacterial infection such as* P. aeruginosa* pneumonia, there are interesting* in vitro* data, albeit not directly applicable in our case [27]. B cells and immunoglobulins may promote the diversity and function of T cells through effects on the T cell receptor diversity in T cell precursors in the thymus [27].

There is another interesting point regarding the degree of lung injury associated with PJP and our patient's low CD8+ T cells on presentation that increased only after hospital discharge.* In vitro* data by Gigliotti et al. [28] indicate that although CD8+ T cells may not play a significant role in control of* P. jiroveci* replication they consist a key component of the inflammatory response and consequent lung injury induced by the organism. The authors suggest that treatment strategies to blunt or eliminate the CD8+ T cell mediated inflammatory response to* P. jiroveci*—an event that probably happened spontaneously in our case due to the nature of the underlying immunodeficiency—combined with antibiotic treatment should result in improved lung function without an adverse impact on clearance of the organism [28].

## 4. Conclusion

In conclusion, this case showed particular importance in several aspects. It illustrated that PJP can rarely be the initial clinical presentation of CVID and particularly LOCID, a newly described CVID subgroup in adults. It presented several diagnostic problems and especially the requirement for differential diagnosis of CVID/LOCID from combined immunodeficiency and idiopathic CD4 lymphocytopenia. Finally, the bouts of improvement and deterioration of the lung injury made us implicate immunoglobulin substitution in both immune reconstitution phenomena and ultimate recovery of both cellular and humoral immunities.

## Figures and Tables

**Table 1:** Immunoglobulin levels during hospital stay and follow-up.

	Hospital stay				Follow-up		
	14/08/06	06/09/06*	04/10/06*	29/11/06	5/06/07**	4/09/07**	15/12/08**
IgG (n.l: 7–17g/L)	3.90	12.90	9.47	15.10	6.10	4.80	9.38
IgM (n.l: 0.4–2.8 g/L)	0.26	0.86	1.36	0.40	0.07	0.06	0.06
IgA (n.l: 0.7–4 g/L)	2.96	4.00	1.76	2.31	3.30	2.15	3.68

*Measured after a 5-day immunoglobulin infusion course.

**In the later three values shown the patient was on regular follow-up and maintained on immunoglobulin replacement with subcutaneous injections. Immunoglobulin levels were trough levels; they were drawn just before the next injection.

n.l: normal limits.

**Table 2:** Measurements of lymphocyte subpopulations via flow cytometry during hospital stay and at follow-up.

	Hospital stay		Follow-up		
	13/10/2006	22/11/2006	15/06/2007	27/09/2007	15/12/2008
WBC (lower cut-off: >4000 cells/*μ*L)	2200	3920	4250	n/a	5000
Lymphocytes (lower cut-off: >1000 cells/*μ*L)	320	510	720	n/a	740
CD4+ T (n.l: 663–1477 cells/*μ*L, 42–58%)	52/16.4%	46/9.0%	46/6.34%	4.6%	23/3.1%
CD8+ T (n.l: 342–754 cells/*μ*L, 22–30%)	26/8.0%	337/66%	466/64.5%	70.3%	555/74.9%
CD4/CD8 ratio	2.0	0.1	0.1	<0.1	<0.1
CD3+ T (n.l: 1500–1900 cells/*μ*L, 69.5–81.3%)	82/25.5%	357/70%	486/67.5%	77.5%	575/77.7%
CD19+ B (n.l: 150–400 cells/*μ*L, 6.80–16.4%)	5/1.5%	5/1%	103/14.3%	8.5%	71/9.6%
NK (n.l: 100–300 cells/*μ*L, 3.30–12.5%)	192/60.3%	77/15%	311/43.2%	19.9%	78 /10.5%
CD4+ CD45RA+ T	n/a	0.27%	0.4%	n/a	n/a
CD4+ CD45RO+ T	n/a	8.5%	5.2%	n/a	n/a

WBC: white blood cells, CD4+ CD45RA+ T: naïve CD4+ T lymphocytes, CD4+ CD45RO+ T: memory CD4+ T lymphocytes, n/a: not available, and n.l: normal limits.

CD4+ T and CD8+ T, CD3+ T, CD19+ B, and NK lymphocytes are depicted in absolute values and percentage (%) of blood lymphocytes.

CD4+ CD45RA+ T and CD4+ CD45RO+ T lymphocytes were calculated in the total number of lymphocytes of the sample for which data is shown.
